# Comprehensive detection of *CRLF2* alterations in acute lymphoblastic leukemia: a rapid and accurate novel approach

**DOI:** 10.3389/fmolb.2024.1362081

**Published:** 2024-02-02

**Authors:** José Vicente Gil, Alberto Miralles, Sandra de las Heras, Esperanza Such, Gayane Avetisyan, Álvaro Díaz-González, Marta Santiago, Carolina Fuentes, José María Fernández, Pilar Lloret, Irene Navarro, Pau Montesinos, Marta Llop, Eva Barragán

**Affiliations:** ^1^ Accredited Research Group on Hematology, Instituto de Investigación Sanitaria la Fe, Valencia, Spain; ^2^ Hematology Service, Hospital Universitario y Politécnico la Fe, Valencia, Spain; ^3^ Centro de Investigación Biomédica en Red de Cáncer, CIBERONC CB16/12/00284, Instituto de Salud Carlos III, Madrid, Spain; ^4^ Accredited Research Group on Clinical and Translational Cancer Research, Instituto de Investigación Sanitaria la Fe, Valencia, Spain; ^5^ Onco-Hematology Unit, Pediatrics Service, Hospital Universitario y Politécnico la Fe, Valencia, Spain; ^6^ Molecular Biology Unit, Clinical Analysis Service, Hospital Universitario y Politécnico la Fe, Valencia, Spain

**Keywords:** acute lymphoblastic leukemia, Ph-like ALL, *CRLF2* overexpression, *CRLF2* variants, JAK2 variants, RT-qPCR-SYBR-HRM

## Abstract

**Introduction:** Acute lymphoblastic leukemia (ALL) is a prevalent childhood cancer with high cure rate, but poses a significant medical challenge in adults and relapsed patients. Philadelphia-like acute lymphoblastic leukemia (Ph-like ALL) is a high-risk subtype, with approximately half of cases characterized by *CRLF2* overexpression and frequent concomitant *IKZF1* deletions.

**Methods:** To address the need for efficient, rapid, and cost-effective detection of *CRLF2* alterations, we developed a novel RT-qPCR technique combining SYBR Green and highresolution melting analysis on a single plate.

**Results:** The method successfully identified *CRLF2* expression, *P2RY8::CRLF2* fusions, and *CRLF2* and *JAK2* variants, achieving a 100% sensitivity and specificity. Application of this method across 61 samples revealed that 24.59% exhibited *CRLF2* overexpression, predominantly driven by *IGH::CRLF2* (73.33%). High Resolution Melting analysis unveiled concurrent *CRLF2* or *JAK2* variants in 8.19% of samples, as well as a dynamic nature of *CRLF2* alterations during disease progression.

**Discussion:** Overall, this approach provides an accurate identification of *CRLF2* alterations, enabling improved diagnostic and facilitating therapeutic decision-making.

## 1 Introduction

Acute lymphoblastic leukemia (ALL) is the most common childhood cancer, accounting for approximately 25% of all pediatric malignancies. In adults, ALL is far less frequent, but it represents a severe disease where only 30%–40% of patients achieve long-term remission rates (Terwilliger and Abdul-Hay, 2017). Despite advances in current therapies, relapse remains a major clinical challenge, emphasizing the importance of identifying genomic alterations associated with disease progression and therapeutic resistance (Advani, 2022; Lejman et al., 2022).

**FIGURE 1:**
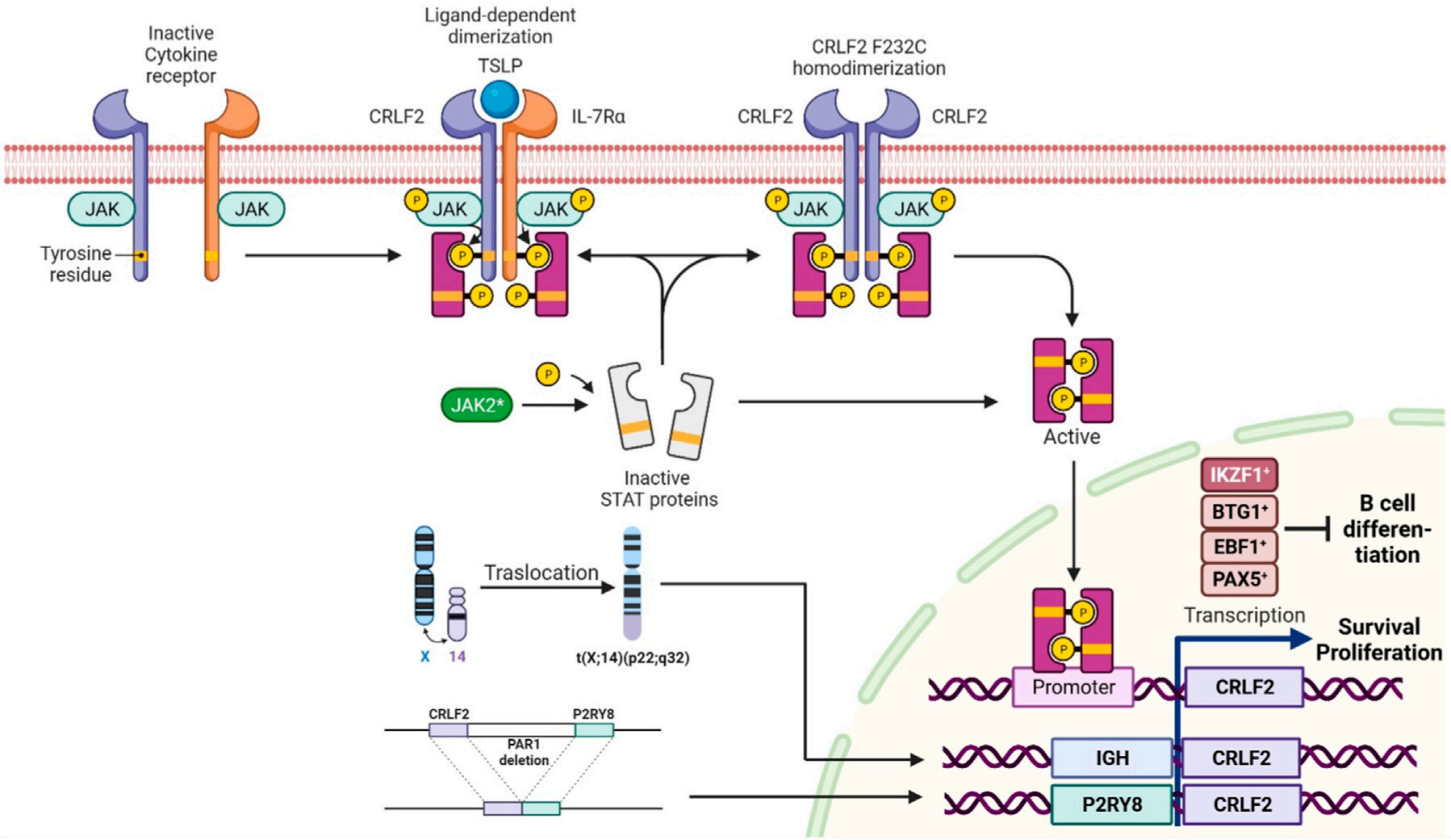
Normal activation of CRLF2 occurs upon the binding of thymic stromal lymphopoietin (TSLP), which induces a conformational change resulting in receptor dimerization. *CRLF2* rearrangements, p.F232C SNV, and *JAK2* variants promote STAT5 phosphorylation and JAK-STAT pathway activation, leading to oncogenic CRLF2 overexpression and aberrant signaling activation. Deletions of B-lymphoid transcriptional regulators, such as *IKZF1, EBF1, BTG1*, and *PAX5*, also upregulate *CRLF2*, leading to disrupted cell differentiation. The image was created in Biorender (https://biorender.com/).

Philadelphia-like ALL (Ph-like ALL) is a high-risk B-ALL subtype that affects 15%–30% of older children and adolescents/young adults (AYAs). This entity is characterized by a gene expression profile resembling Ph + ALL, but lacks the t (9; 22) (q34; q11) chromosomal translocation. Instead, it exhibits multiple genetic alterations converging in tyrosine kinase and cytokine receptor signaling pathways, such as *ABL*-class fusions, *JAK2* rearrangements or *CRLF2* alterations (Roberts, 2017). Approximately 50% of Ph-like ALL manifest *CRLF2* lesions, with overexpression being a hallmark driven by different mechanisms, including 1) *P2RY8::CRLF2* fusion (due to a cryptic deletion within the pseudoautosomal region 1 (PAR1) of the sex chromosomes, which juxtaposes CRLF2 to the P2RY8 promoter; 2) *CRLF2* translocation at *IGH* locus, positioning *CRLF2* under the control of the immunoglobulin promoter; 3) *CRLF2* single-nucleotide variants (SNVs; with p.F232C as the most frequent); and 4) other JAK-STAT pathway activating variants (i.e., *JAK2* p.R683G) (Roberts et al., 2018). Moreover, concomitant *IKZF1* deletions are frequent in Ph-like ALL, further underscoring the intricate genetic landscape of this recently recognized entity (Russell et al., 2009; Harvey et al., 2010a) (Figure 1).

The potential use of targeted therapies, such as Bruton’s tyrosine kinase (BTK) inhibitors for patients with *IKZF1* deletions or ruxolitinib for those with *CRLF2* rearrangements (*CRLF2-r*) or JAK-STAT pathway lesions, has been explored (Tasian et al., 2018; Holmes et al., 2019; Yu et al., 2022). However, some studies have shown limited effectiveness of ruxolitinib as single agent as well as inferior outcome in *CRLF2-r* with concomitant JAK variant cases compared to *CRLF2-r* patients (Roberts et al., 2014; Bӧhm et al., 2021). This scenario remarks the relevance of *CRLF2* overexpression identification, as well as the underlying molecular mechanisms for ALL diagnosis and treatment selection. Furthermore, it could be useful during patient follow-up to identify clonal evolution, adapt therapy, and prevent treatment failure (Palmi et al., 2016; Wang et al., 2023).

There is no consensus on the methodology to characterize *CRLF2* alterations. Most approaches rely on high-throughput technologies (NGS, low density arrays, etc.), which may not be available in all laboratories and, in some cases, the causative mechanisms behind *CRLF2* overexpression may not be fully elucidated. Real-time PCR-based techniques are broadly available, cost-effective and versatile and offer a short turnaround time. High Resolution Melting (HRM) and SYBR Green fluorescent detection are commonly used in diagnostic laboratories for genotyping, single nucleotide variation (SNV) detection and gene expression assessment, respectively, so they could represent a valid approach for *CRLF2* study. However, there remains a notable gap in the literature regarding methods that integrate both approaches to identify the different molecular alterations affecting *CRLF2* expression.

In the present study, we delineate a novel comprehensive, fast, and cheap technique combining HRM analysis and SYBR Green dye (RT-qPCR-SYBR-HRM), allowing for the detection of *CRLF2* expression and the main causative mechanisms in a single experiment. We demonstrate that the method is sensible, specific, convenient, and versatile for this purpose.

## 2 Materials and methods

### 2.1 Patients

The study encompassed two patient cohorts diagnosed at Hospital Universitari i Politècnic La Fe (Valencia, Spain). Cohort A comprised 100 retrospective ALL patients: 74 pediatric (0–17 years), 12 AYA (18–42 years) and 14 adult (>42 years) patients (Supplementary Table S1). Cohort B consisted of 61 prospective samples, including 46 pediatric, 11 AYA, and 4 adult patients (Supplementary Table S2). The inclusion criteria for this study were as follows: availability of high-quality DNA and/or RNA obtained from bone marrow or peripheral blood, and documented written consent obtained in compliance with the recommendations of the Declaration of Human Rights and the Helsinki Conference. This study was approved by the institutional ethics committee for clinical research.

All patients were initially characterized using the following methods: karyotyping and fluorescence *in situ* hybridization (FISH, including *CRLF2* rearrangement status) were performed according to standard protocols. *ETV6::RUNX1* and *BCR::ABL1* fusions were assessed by RT-qPCR (Gabert et al., 2003).

### 2.2 Nucleic acid extraction and quantitation

Genomic DNA or RNA from bone marrow or peripheral blood was isolated using the QIAamp DNA Midi Kit or the RNeasy Midi Kit (Qiagen, Valencia, CA), respectively, using the QIAsymphony SP/AS instrument (Qiagen) according to manufacturer’s instructions. The quantification and quality of nucleic acids were assessed with a QIAxpert instrument (QIAgen). Reverse transcription was performed on 500 ng of RNA using the SuperScript IV VILO kit (Thermo Fisher Scientific, San Francisco, CA, United States).

### 2.3 Development of a new RT-qPCR-SYBR-HRM method for *CRLF2* expression assessment and the identification of the underlying molecular mechanisms

#### 2.3.1 Method approach

**FIGURE 2:**
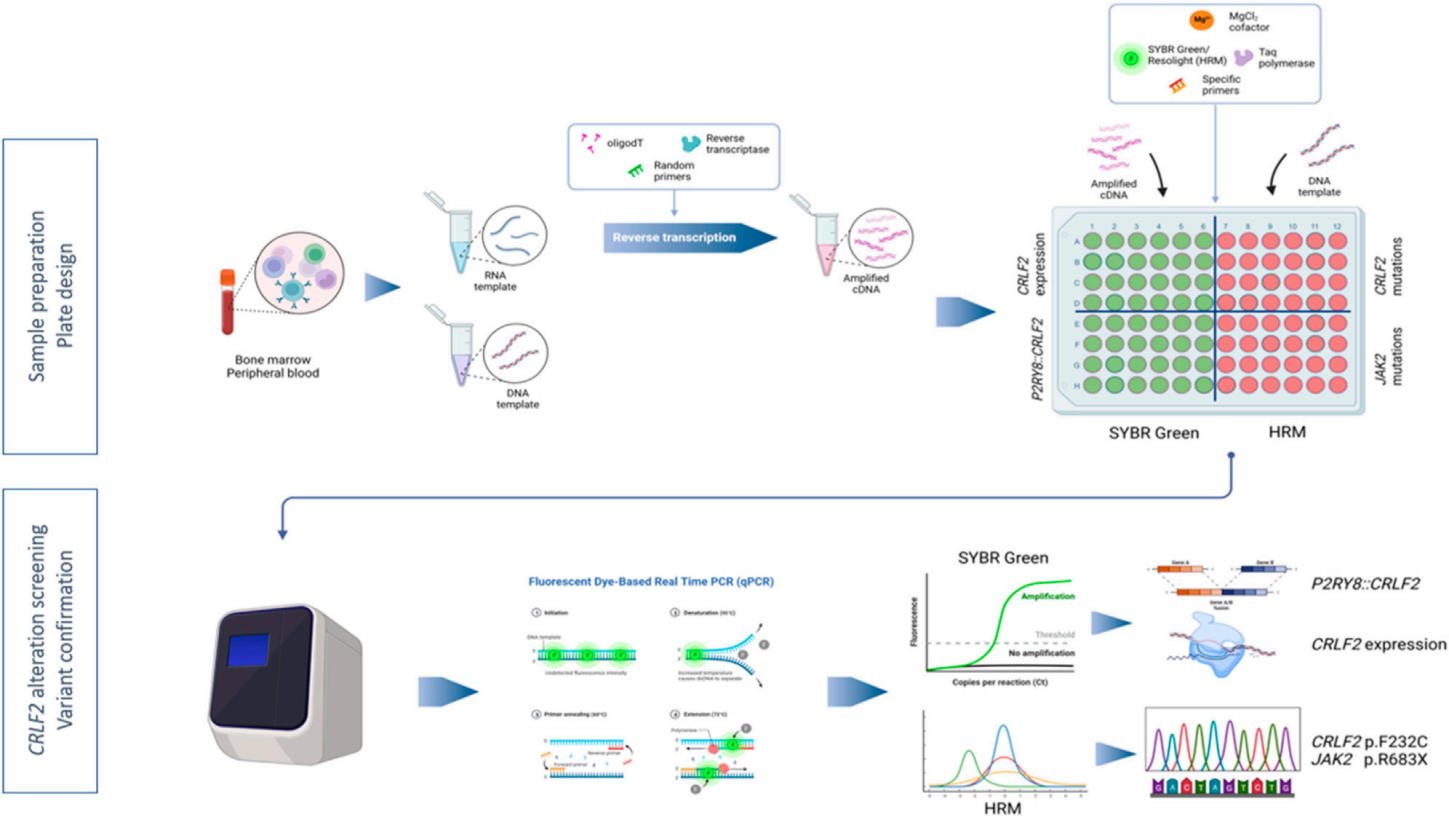
Workflow depicting the RT-qPCR-SYBR-HRM method to assess *CRLF2* alterations. Nucleic acids were isolated from bone marrow or peripheral blood. RNA was subsequently subjected to reverse transcription to obtain cDNA. Real-time PCR plate was set up in order to assess, in parallel, *CRLF2* expression and fusions (SYBR Green fluorescent detection) and *CRLF2* and *JAK2* pathogenic variants (HRM analysis). The image was created in Biorender (https://biorender.com/).

A novel RT-qPCR-SYBR-HRM approach was developed in order to study *CRLF2* expression and to identify the underlying molecular mechanisms, using a common cycling protocol: in the same plate, using cDNA as template and SYBR Green, *CRLF2* expression and *P2RY8::CRLF2* presence were assessed; in parallel, *CRLF2* and *JAK2* screening was performed by HRM using patients’ DNA (Figure 2).

All experiments were carried out on a LightCycler480 Instrument (Roche Diagnostics, Rotkreuz, Switzerland). The thermal cycling protocol included an initial 5-min hold at 40°C for UDG activation, followed by an 8-min hold at 95°C. The PCR steps consisted of 10 s at 95°C, 15 s at 58°C, and 30 s at 72°C for 45 cycles. Melting analysis was performed with a final denaturalization (95°C for 1 min) and cooling (40°C for 1 min), and a 60°C–95°C melting gradient with a ramp rate of 0.04°C/s and continuous acquisition mode set at 25 acquisitions/°C.

#### 2.3.2 *CRLF2* expression

To assess *CRLF2* expression, SYBR Green and specific primers were used (Supplementary Table S1). All experiments were conducted in duplicate, each in a 10 µL reaction containing 1U Uracil-DNA glycosylase (UDG) (Thermo Fisher Scientific), 1.2 mM of each primer, 1X SYBR Green Master Mix (Roche), and 2 μL of cDNA. *CRLF2* expression was quantified using the 2^−ΔΔCT^ method, using median expression from cohort A as a reference value, and *ABL1* as the housekeeping gene. The ΔΔCts were calculated by subtracting the median of the ΔCt of the cohort A tested patients to the ΔCt of each sample.

##### 2.3.2.1 *CRLF2* overexpression criteria

To accurately identify differences in gene expression and establish a reliable overexpression criterion, *CRLF2* expression was assessed in a retrospective cohort of 100 ALL patients (cohort A). The 20-fold interquartile range (IQR) was considered as the overexpression cut off value (CRLF2^IQR20^). This threshold, expressed as 2^−ΔΔCt^ relative to the median, was 120.19.

#### 2.3.3 P2RY8::CRLF2 detection

*P2RY8::CRLF2* fusion was assessed as described in Section 2.3.2 using specific primers (Supplementary Table S3).

#### 2.3.4 *CRLF2* and *JAK2* variants

HRM analysis was performed on *CRLF2* (exon 6) and *JAK2* (exon 16) hotspots. All samples were tested in duplicate, positive and negative controls for each exon were included in each run. PCR was carried out using 40 ng of genomic DNA, 0.3 µM of each primer (Supplementary Table S3), 3 mM of MgCl_2_, and 1X LightCycler 480 High Resolution Melting Master Mix (Roche). Melting curves were analyzed using Gene Scanning software (Roche). All samples showing divergent melting curves were sequenced following standard protocols on a SeqStudio Genetic Analyzer (Applied Biosystems, Foster City, CA).

#### 2.3.5 Analytical performance

##### 2.3.5.1 Variant limit of detection

The Limit of detection (LoD) was assessed using positive samples for which the variant allele frequency (VAF) was established with Sanger sequencing and the Minor Variant Finder (MVF) software. Samples carrying *CRLF2* and *JAK2* hotspot variants were serially diluted into a negative control, to obtain VAFs of 30%, 15%, 10%, and 5%.

#### 2.3.6 Sensitivity and specificity

Sensitivity [true-positive (TP)/(TP + false-negative (FN))] and specificity [true negative (TN)/(TN + false-positive (FP))] were calculated according to the following definitions: True positive (TP) refers to a known alteration that is expected to be present, and it has been correctly identified. False positive (FP) applies to a variant mistakenly identified. True negative (TN) concerns to a region of interest where the absence of variants is correctly identified. False negative (FN) refers to a missed variant that is expected to be present.

For *CRLF2*-r identification, FISH was considered as the gold standard method. For SNV assessment, Sanger sequencing and the MVF software was used as the reference method.

### 2.4 *IKZF1* deletions

Deletions in *IKZF1* and other genes in ALL (*BTG1*, *CDKN2A/B*, *EBF1*, *ETV6*, PAR1 region, *PAX5* and *RB1*) were investigated by multiple ligation-dependent probes amplification (MLPA) using SALSA MLPA P335 ALL-IKZF1 probemix kit (MRC-Holland, Amsterdam, the Netherlands) according to the manufacturer’s instructions. Four negative controls from healthy donors were included in each run. Capillary electrophoresis was carried out in SeqStudio Genetic Analyzer and data were analyzed using Coffalyser software (MRC-Holland).

### 2.5 Statistical analysis

Medians of quantitative variants were compared with Mann–Whitney’s *U* test. Categorical variables were examined with the chi-square’s test or Fisher’s exact test. Statistical tests were performed with the RStudio software version 4.2.2, and *p* < 0.05 was considered statistically significant. Plots were generated using ggplot2 package version 3.4.2, Fishplot package v0.5 and cBioPortal, a free web-based platform for interactive data visualization.

## 3 Results

### 3.1 Technical validation

#### 3.1.1 Sensitivity, specificity and limit of detection

A high concordance between the RT-qPCR-SYBR-HRM method and the gold standard techniques was obtained, with a sensitivity and specificity of 100% for all alterations (Table 1).

**TABLE 1:** Sensitivity and specificity achieved by the RT-qPCR-SYBR-HRM method.

Method	Target	Sensitivity, % [TP/(TP + FN)]	Specificity, % [TN/(TN + FP)]
RT-qPCR vs. FISH	*CRLF2* overexpression^a^	100	100
	*P2RY8::CRLF2*	100	100
HRM vs. Sanger	*CRLF2*	100	100
	*JAK2*	100	100

^a^ We assumed that virtually all CRLF2-r samples overexpress this gene.

The lowest VAF detected by HRM was found at a dilution of 1:6 for *CRLF2* p.F232C and *JAK2* p.R683G variants, corresponding to a 5% VAF (Supplementary Figure S1).

### 3.2 *CRLF2* alterations detected in ALL patients

#### 3.2.1 *CRLF2* expression, fusions and activating hotspot variants

In total, 61 samples from 56 patients (cohort B) were prospectively analyzed with the method. Patients’ main characteristics are shown in Supplementary Table S2.

*CRLF2* expression levels ranged from 0.006- to 1704.34-fold expressed as 2^−ΔΔCt^. Median *CRLF2* expression did not significantly differ between cohort A and B (3.81 vs. 6.19; *p* = 0.43).

**FIGURE 3:**
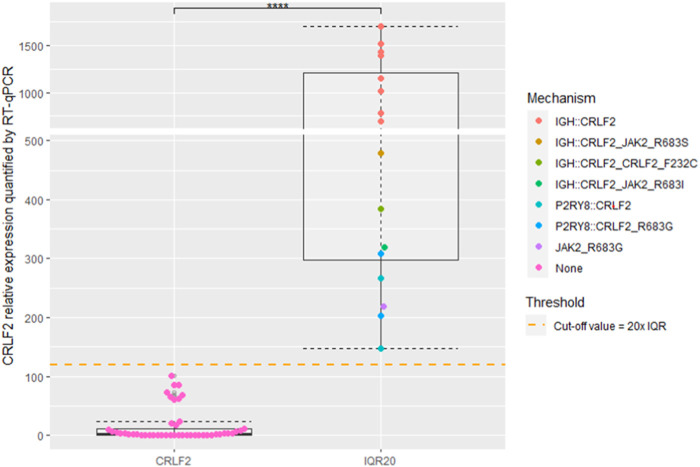
Box and Whisker plots depicting *CRLF2* expression. Patients were categorized into two distinct groups based on their expression levels: *CRLF2* and *CRLF2* ^IQR20^. *CRLF2* ^IQR20^ cutoff value is indicated with an orange dashed line. Color-coded dots indicate the assessed mechanisms inducing CRLF2 overespression. **** indicate a *p*-value <0.001.

*CRLF2* ^IQR20^ was identified in 15/61 (26.2%) samples. The molecular upregulation mechanism was identified in 15/15 (100%) samples. Eleven patients (11/15, 73.3%) carried the *IGH::CRLF2* fusion (detected by FISH), 3/15 (20%) harbored the *P2RY8::CRLF2* fusion, and 1/15 (6.7%) carried the *JAK2* p.R683G variant. Five out of 14 (35.7%) *CRLF2-r* samples carried concomitant *CRLF2* (p.F232C or *JAK2* (2 p.R683G, 1 p.R683S, 1 p.R683I) point variants (Figure 3).

Of note, 8/46 not-overexpressing samples showed a slightly higher *CRLF2* expression that clustered between IQR10 and IQR20 values. The method identified the *P2RY8::CRLF2* fusions at subclonal levels (not identified by FISH) in 2 of these samples (25%) (Supplementary Figure S2).

The median age in *CRLF2-r* patients was significantly higher than that in non-rearranged ALL (27.5 vs. 9.26, *p* < 0.001). *CRLF2* expression was significantly higher in *IGH::CRLF2* patients compared to *P2RY8::CRLF2* patients (median 1,156.1 vs. 234.6, *p* < 0.005). No significant differences in *CRLF2* expression were observed among other demographic or clinical variables. A comprehensive summary of the results is provided in Supplementary Table S4.

### 3.3 *IKZF1* and other gene deletions

*IKZF1* deletions were significantly (*p* < 0.01) more frequent in *CRLF2* ^IQR20^ patients: 7/15 *CRLF2* ^IQR20^ (46.67%; 4 *IGH::CRLF2* and 3 *P2RY8::CRLF2* samples) vs. 5/46 no *CRLF2* ^IQR20^ patients (10.87%).

**FIGURE 4:**
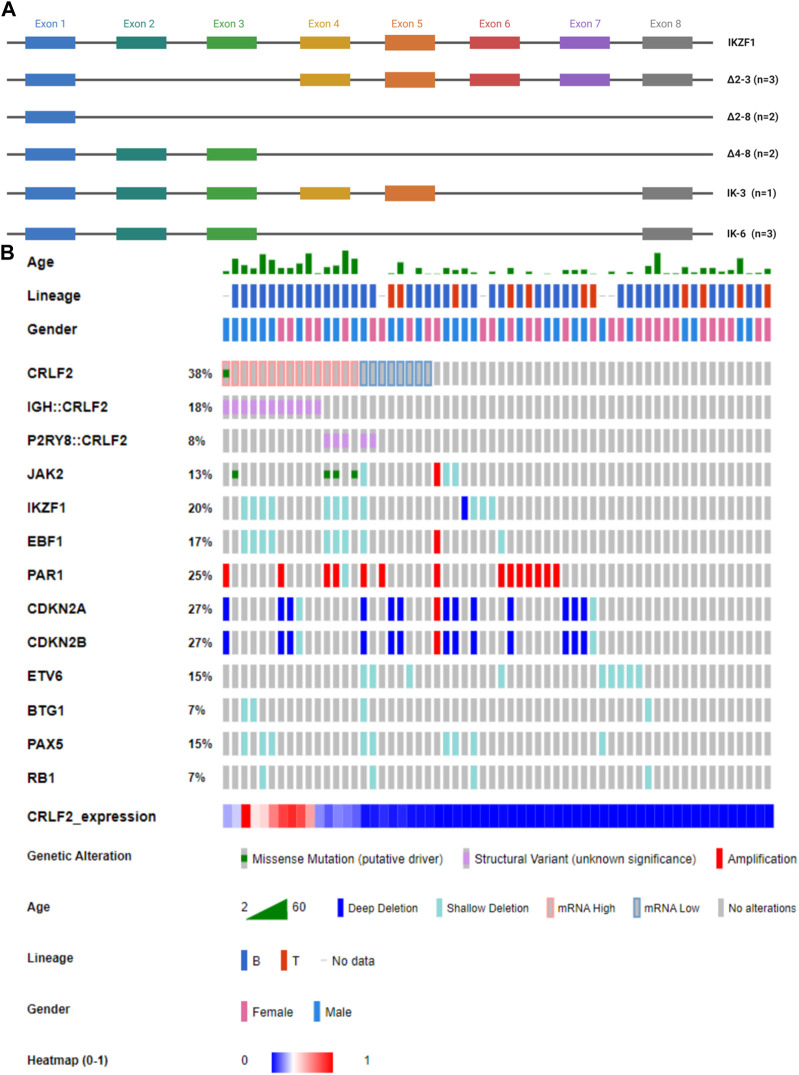
**(A)** Schematic representation of *IKZF1* isoforms found in our cohort through MLPA analysis. The colored boxes indicate distinct exons. IK-6 is a dominant-negative isoform, whereas ∆2–3, ∆two to eight and ∆four to eight isoforms cause haploinsufficiency. **(B)** Oncoprint showing *CRLF2* alterations with additional CNVs. The Oncoprint also includes demographic and clinical data of the patients.

In our study cohort, a deletion of exons two to eight in *IKZF1*, denoted as Δ2-8, was found in a patient with the *P2RY8::CRLF2* fusion (Figure 4). Additionally, Δ4-8 alterations were identified in two patients carrying the *IGH::CRLF2* and another with the *P2RY8::CRLF2* fusion. Furthermore, three patients exhibited the Δ2-3 isoform, of which two had the *IGH::CRLF2* fusion, while the third patient tested negative for *CRLF2*-r. One patient with subclonal *P2RY8::CRLF2* fusion showed the IK-3 and IK-6 isoforms, whereas two *CRLF2*-r negative patients carried the IK-6 isoform (Figure 4A).

Eighty-nine additional CNVs were identified in 42/61 (68.85%) samples. The mean number of affected genes per patient was 1.67, range 0–9. The most frequently altered genes were *CDKN2A* (26.23%), *CDKN2B* (26.23%), and the PAR1 region (24.59%), as shown in Figure 4B and Supplementary Table S4. In the AYA subgroup, a co-occurrence pattern (*p* < 0.001) was observed between *EBF1* and *IKZF1* deletions, whereas in the pediatric subgroup, this association was not significant. Notably, no mutually exclusive genetic alterations were identified in our cohort. No significant differences were found in terms of *CRLF2* expression.

### 3.4 Clonal evolution of *CRLF2* alterations

To investigate whether *IGH::CRLF2* and *P2RY8::CRLF2* were early or late events during leukemogenesis, we further analyzed *CRLF2*-r samples (*IGH::CRLF2*, *n* = 11 and *P2RY8::CRLF2*, *n* = 3) according to the tumoral load and disease stage. In patients harboring *IGH::CRLF2* at diagnosis, the blast counts correlated with the percentage of rearranged cells detected by FISH, suggesting that *IGH::CRLF2* was present in the major leukemic clone. In contrast, *P2RY8::CRLF2* fusion constituted a minor clone among the blast count, thus confirming its secondary nature. Of note, subclonal levels of *P2RY8::CRLF2* were detected in two patients, one carrying *ETV6::RUNX1* as the primary leukemic event, and another harboring an *IKZF1* deletion (IK-3) and other CNVs (Supplementary Table S4).

**FIGURE 5:**
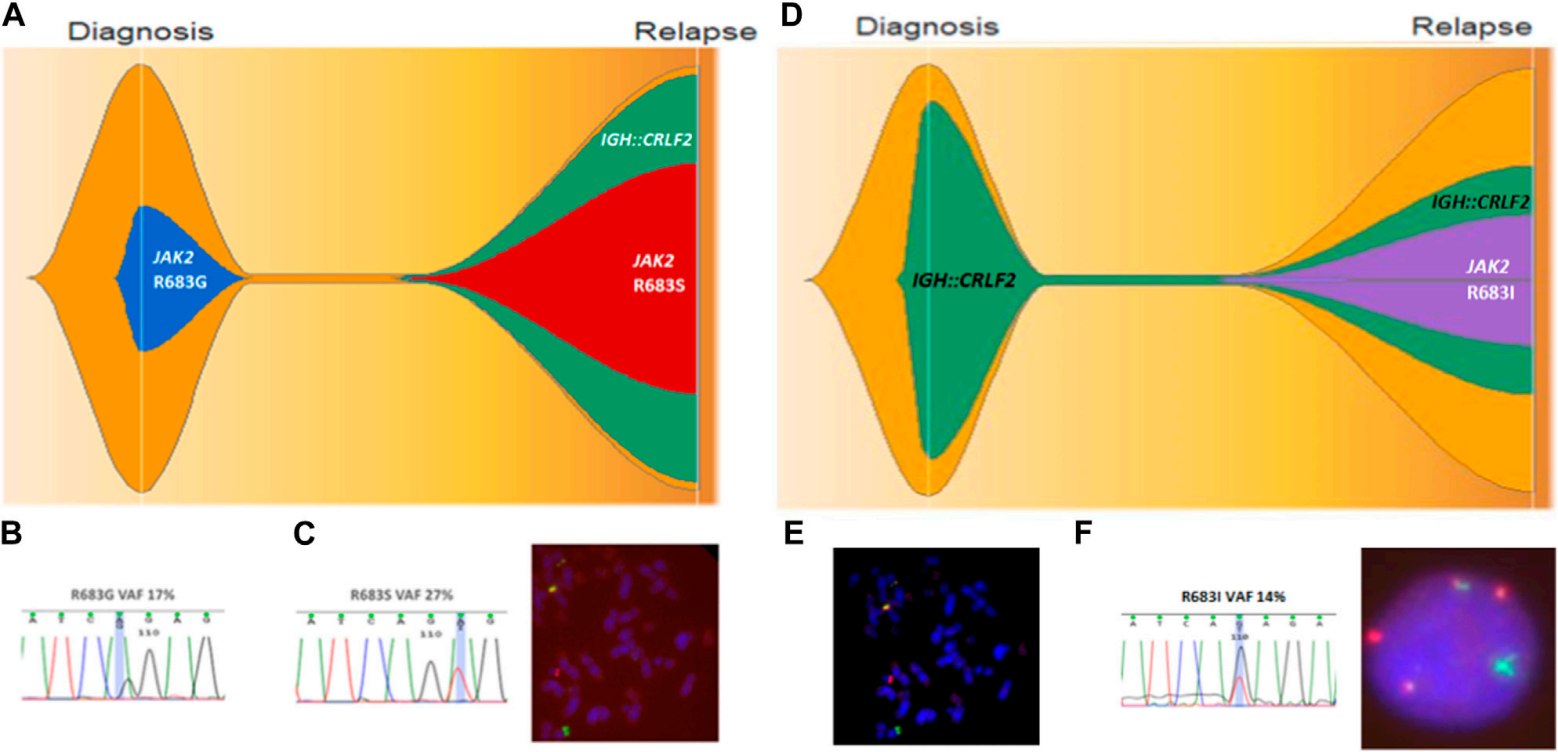
Clonal evolution of patient 2 **(A–C)** and patient 12 **(D–F)**. **(A)** Fish plot showing clonal evolution where *JAK2* p.R683G is lost at relapse and *IGH::CRLF2* and *JAK2* p.R683S are acquired. **(B)** *JAK2* p.R683G confirmed by direct sequencing with 17% VAF. **(C)** *JAK2* R683S confirmed by Sanger sequencing and *IGH::CRLF2* fusion identified by FISH at relapse. **(D)** Fish plot showing clonal evolution where *JAK2* p.R683I is acquired at relapse. **(E)** *IGH::CRLF2* fusion identified at diagnosis by FISH. **(F)** *IGH::CRLF2* fusion and *JAK2* p.R683I identified at relapse.

Paired diagnosis-relapse samples could be analyzed in 4 *CRLF2* ^IQR20^ patients, among whom 2/4 (50%) experienced clonal evolution. Patient 2 harbored the *JAK2* p.R683G variant at diagnosis, which was lost at relapse but acquired both *IGH::CRLF2* and *JAK2* p.R683S variant, resulting in a 2-fold *CRFL2* expression increase compared to diagnosis (Figures 5A–C). A different evolution pattern was observed in Patient 12, who carried *IGH::CRLF2* at diagnosis and acquired a *JAK2* variant (p.R683I) at relapse, reducing *CRLF2* expression 3-fold compared to diagnosis (Figures 5D–F).

## 4 Discussion

In this study, we develop a RT-qPCR-SYBR-HRM method able to quantify *CRLF2* expression and detect the main underlying deregulation mechanisms such as the *P2RY8::CRLF2* fusion and *CRLF2* and *JAK2* pathogenic variants.

The approach showed 100% sensitivity and specificity for the identification of *CRFL2*-r patients, and the same accuracy was found for the detection of *P2RY8::CRLF2* and *CRLF2* and *JAK2* variants. The 5% VAF limit of detection established for SNVs is widely accepted in the clinical context (Richards et al., 2015; Pandzic et al., 2022), and lower than that generally attributed to direct Sanger sequencing.

High throughput alternatives like NGS, Low Density Arrays or Optical Genome Mapping constitute powerful tools to assess clinically relevant alterations in a single experiment; but its use may be limited by their long turnaround time, as the identification of patients eligible for targeted therapies requires rapid results (Malone et al., 2020). Moreover, the implementation of high-throughput techniques may be challenging in low-income countries due to the need of complex validation studies, logistic challenges and technical expertise (Alonso et al., 2019; Llop et al., 2022; Gil et al., 2023). Thus, conventional methods still play an important role in molecular laboratories as they provide sensitive, reliable, and cost-effective results. In contrast with other published RT-qPCR-based methods, our approach provides a comprehensive study of *CRLF2* characterized by its flexibility, rapidity, and low cost (Shochat et al., 2011; Palmi et al., 2012).

In our exploratory cohort 24.59% samples showed *CRLF2* ^IQR20^ expression. In line with Palmi et al., 2016, the driving mechanism was *IGH::CRLF2* in 73.3% of patients and *P2RY8::CRLF2* in 20%. Moreover, subclonal *P2RY8::CRLF2* was found in 2/61 samples. Previous research has shown that subclonal *P2RY8::CRLF2* represents a secondary event during leukemogenesis, usually lost at relapse (Morak et al., 2012; Vesely et al., 2016). Our results further support this hypothesis, as subclonal *P2RY8::CRLF2* samples carried other driver fusions (*ETV6::RUNX1* and *PAX5*-r).This data underscores the lack of clinical significance associated with subclonal *CRLF2* alterations and supports the accuracy of the selected overexpression criteria.

Only one patient harboured a SNV in *CRLF2* (1.79%) whereas *JAK2* variants were found in 5.36% patients. This could be explained by the reported low frequency of *CRLF2* pathogenic variants in pediatric cohorts, which is approximately 2% (Chen et al., 2012), and the higher co-occurrence (about 10%) between *JAK2* variants and *CRLF2*-r in non-Down Syndrome ALL (Harvey et al., 2010b). Previous studies interrogating *CRLF2* and *JAK2* in ALL have shown that variants can also be found outside the hotspots screened. However, their frequency is very low and their clinical relevance is not well established (Mullighan and Downing, 2009; Harvey et al., 2010b).

In agreement with Russell et al. (2017), AYAs showed higher *CRLF2* expression than pediatric patients, which could be explained by the fact that median age was significantly higher in *CRLF2*-r patients. Moreover, within *CRLF2*-r patients, the ones harbouring *IGH::CRLF2* were significantly older that those with *P2RY8::CRLF2*. We did not find any significant associations between *CRLF2* expression and other demographic and clinical variables.

As previously described, we observed *IKZF1* deletions in a substantial proportion of *CRLF2* ^IQR20^ samples (46.67%), a genetic trait associated with a high relapse rate and poor overall survival (Stanulla et al., 2020). Furthermore, we identified several *IKZF1* isoforms in our patient cohort, suggesting that any alteration affecting IKAROS function could be involved in leukemogenesis as mentioned by Conserva et al. (2023). In contrast to Russell et al. (2017), we could not find significant differences regarding *IKZF1* or *BTG1* deletions in *CRLF2-r* patients, probably due to the low size of our cohort. We also observed a significant association between *EBF1* and *IKZF1* deletions in the AYAs subgroup, but not in pediatric patients. This association has been previously described by different authors, who suggest that *EBF1* and *IKZF1* cooperate in B cell differentiation blockage and higher minimal residual disease levels compared to isolated *IKZF1* deletions (Mullighan et al., 2009; Marke et al., 2018; Steeghs et al., 2019).

Clonal evolution was observed in *CRLF2* ^IQR20^ patients. These findings are clinically relevant, as patients with *CRLF2-r* and concomitant *JAK2* variants contribute to inferior outcomes in ALL (Jain et al., 2017). In agreement with Palmi et al. (2012), we observed that *P2RY8::CRLF2* is a secondary event in *CRLF2-r* patients. In this context, our results suggest a dynamic nature of *CRLF2* alterations during disease progression. Monitoring genetic markers enables clinicians to tailor treatment strategies, adapt interventions to evolving genomic abnormalities, and minimize the risk of treatment failure (Iacobucci and Mullighan, 2017). Furthermore, identifying clonal evolution can be decisive for the selection of appropriate therapeutic approaches, including targeted therapies addressing specific genetic alterations acquired during disease progression (Sayyab et al., 2021).

In conclusion, we developed a new versatile, sensitive, and cheap tool to identify and characterize *CRLF2* deregulated patients, which can assist the therapeutic decision-making. These findings contribute to a better understanding of the pathogenesis of Ph-like ALL with *CRLF2* alterations.

## Data Availability

The original contributions presented in the study are included in the article/Supplementary Material, further inquiries can be directed to the corresponding author.

## References

[B1] Advani A. S. (2022). Novel strategies in the treatment of acute lymphoblastic leukaemia. Lancet. Haematol. 9 (4), e240–e241. 10.1016/s2352-3026(22)00054-0 35358432

[B2] Alonso C. M. Llop M. Sargas C. Pedrola L. Panadero J. Hervás D. (2019). Clinical utility of a next-generation sequencing panel for acute myeloid leukemia diagnostics. J. Mol. diagnostics JMD 21 (2), 228–240. 10.1016/j.jmoldx.2018.09.009 30576870

[B4] Bӧhm J. W. Sia K. C. S. Jones C. Evans K. Mariana A. Pang I. (2021). Combination efficacy of ruxolitinib with standard-of-care drugs in CRLF2-rearranged Ph-like acute lymphoblastic leukemia. Leukemia 35 (11), 3101–3112. 10.1038/s41375-021-01248-8 33895784

[B5] Chen I.-M. Harvey R. C. Mullighan C. G. Gastier-Foster J. Wharton W. Kang H. (2012). Outcome modeling with CRLF2, IKZF1, JAK, and minimal residual disease in pediatric acute lymphoblastic leukemia: a Children’s Oncology Group study. Blood 119 (15), 3512–3522. 10.1182/blood-2011-11-394221 22368272 PMC3325039

[B6] Conserva M. R. Redavid I. Anelli L. Zagaria A. Tarantini F. Cumbo C. (2023). IKAROS in acute leukemia: a positive influencer or a mean hater? Int. J. Mol. Sci. 24 (4), 3282. 10.3390/ijms24043282 36834692 PMC9961161

[B7] Gabert J. Beillard E. van der Velden V. H. J. Bi W. Grimwade D. Pallisgaard N. (2003). Standardization and quality control studies of ‘real-time’ quantitative reverse transcriptase polymerase chain reaction of fusion gene transcripts for residual disease detection in leukemia - a Europe against Cancer program. Leukemia 17 (12), 2318–2357. 10.1038/sj.leu.2403135 14562125

[B8] Gil J. V. Such E. Sargas C. Simarro J. Miralles A. Pérez G. (2023). Design and validation of a custom next-generation sequencing panel in pediatric acute lymphoblastic leukemia. Int. J. Mol. Sci. 24 (5), 4440. 10.3390/ijms24054440 36901871 PMC10002321

[B9] Harvey R. C. Mullighan C. G. Chen I. M. Wharton W. Mikhail F. M. Carroll A. J. (2010b). Rearrangement of CRLF2 is associated with mutation of JAK kinases, alteration of IKZF1, Hispanic/Latino ethnicity, and a poor outcome in pediatric B-progenitor acute lymphoblastic leukemia. Blood 115 (26), 5312–5321. 10.1182/blood-2009-09-245944 20139093 PMC2902132

[B10] Harvey R. C. Mullighan C. G. Wang X. Dobbin K. K. Davidson G. S. Bedrick E. J. (2010a). Identification of novel cluster groups in pediatric high-risk B-precursor acute lymphoblastic leukemia with gene expression profiling: correlation with genome-wide DNA copy number alterations, clinical characteristics, and outcome. Blood 116 (23), 4874–4884. 10.1182/blood-2009-08-239681 20699438 PMC3321747

[B11] Holmes K. B. Sadreev I. I. Rawstron A. C. Munir T. Westhead D. R. Hillmen P. (2019). Ibrutinib induces chromatin reorganisation of chronic lymphocytic leukaemia cells. Oncogenesis 8 (5), 32. 10.1038/s41389-019-0142-2 31076570 PMC6510766

[B12] Iacobucci I. Mullighan C. G. (2017). Genetic basis of acute lymphoblastic leukemia. J. Clin. Oncol. official J. Am. Soc. Clin. Oncol. 35 (9), 975–983. 10.1200/jco.2016.70.7836 PMC5455679 28297628

[B13] Jain N. Roberts K. G. Jabbour E. Patel K. Eterovic A. K. Chen K. (2017). Ph-like acute lymphoblastic leukemia: a high-risk subtype in adults. Blood 129 (5), 572–581. 10.1182/blood-2016-07-726588 27919910 PMC5290985

[B14] Lejman M. Chałupnik A. Chilimoniuk Z. Dobosz M. (2022). Genetic biomarkers and their clinical implications in B-cell acute lymphoblastic leukemia in children. Int. J. Mol. Sci. 23 (5), 2755. 10.3390/ijms23052755 35269896 PMC8911213

[B15] Llop M. Sargas C. Barragán E. (2022). The role of next-generation sequencing in acute myeloid leukemia. Curr. Opin. Oncol. 34 (6), 723–728. 10.1097/CCO.0000000000000899 36102349

[B17] Malone E. R. Oliva M. Sabatini P. J. B. Stockley T. L. Siu L. L. (2020). Molecular profiling for precision cancer therapies. Genome Med. 12 (1), 8. 10.1186/s13073-019-0703-1 31937368 PMC6961404

[B18] Marke R. van Leeuwen F. N. Scheijen B. (2018). The many faces of IKZF1 in B-cell precursor acute lymphoblastic leukemia. Haematologica 103 (4), 565–574. 10.3324/haematol.2017.185603 29519871 PMC5865415

[B20] Morak M. Attarbaschi A. Fischer S. Nassimbeni C. Grausenburger R. Bastelberger S. (2012). Small sizes and indolent evolutionary dynamics challenge the potential role of P2RY8-CRLF2–harboring clones as main relapse-driving force in childhood ALL. Blood 120 (26), 5134–5142. 10.1182/blood-2012-07-443218 23091296 PMC4194314

[B21] Mullighan C. G. Downing J. R. (2009). Genome-wide profiling of genetic alterations in acute lymphoblastic leukemia: recent insights and future directions. Leukemia 23 (7), 1209–1218. 10.1038/leu.2009.18 19242497

[B22] Mullighan C. G. Zhang J. Harvey R. C. Collins-Underwood J. R. Schulman B. A. Phillips L. A. (2009). JAK mutations in high-risk childhood acute lymphoblastic leukemia. Proc. Natl. Acad. Sci. U. S. A. 106 (23), 9414–9418. 10.1073/pnas.0811761106 19470474 PMC2695045

[B23] Palmi C. Savino A. M. Silvestri D. Bronzini I. Cario G. Paganin M. (2016). CRLF2 over-expression is a poor prognostic marker in children with high risk T-cell acute lymphoblastic leukemia. Oncotarget 7 (37), 59260–59272. 10.18632/oncotarget.10610 27449287 PMC5312310

[B24] Palmi C. Vendramini E. Silvestri D. Longinotti G. Frison D. Cario G. (2012). Poor prognosis for P2RY8-CRLF2 fusion but not for CRLF2 over-expression in children with intermediate risk B-cell precursor acute lymphoblastic leukemia. Leukemia 26 (10), 2245–2253. 10.1038/leu.2012.101 22484421

[B25] Pandzic T. Ladenvall C. Engvall M. Mattsson M. Hermanson M. Cavelier L. (2022). Five percent variant allele frequency is a reliable reporting threshold for TP53 variants detected by next generation sequencing in chronic Lymphocytic leukemia in the clinical setting. HemaSphere 6 (8), e761. 10.1097/HS9.0000000000000761 35935605 PMC9348859

[B28] Richards S. Aziz N. Bale S. Bick D. Das S. Gastier-Foster J. (2015). Standards and guidelines for the interpretation of sequence variants: a joint consensus recommendation of the American college of medical genetics and genomics and the association for molecular pathology. Genet. Med. official J. Am. Coll. Med. Genet. 17 (5), 405–424. 10.1038/gim.2015.30 PMC4544753 25741868

[B29] Roberts K. G. (2017). The biology of Philadelphia chromosome-like ALL. Best Pract. Res. Clin. Haematol. 30 (3), 212–221. 10.1016/j.beha.2017.07.003 29050694

[B30] Roberts K. G. Li Y. Payne-Turner D. Harvey R. C. Yang Y. L. Pei D. (2014). Targetable Kinase-Activating lesions in PH-like acute lymphoblastic leukemia. N. Engl. J. Med. 371 (11), 1005–1015. 10.1056/NEJMoa1403088 25207766 PMC4191900

[B31] Roberts K. G. Reshmi S. C. Harvey R. C. Chen I. M. Patel K. Stonerock E. (2018). Genomic and outcome analyses of Ph-like ALL in NCI standard-risk patients: a report from the Children’s Oncology Group. Blood 132 (8), 815–824. 10.1182/blood-2018-04-841676 29997224 PMC6107876

[B32] Russell L. J. Capasso M. Vater I. Akasaka T. Bernard O. A. Calasanz M. J. (2009). Deregulated expression of cytokine receptor gene, CRLF2, is involved in lymphoid transformation in B-cell precursor acute lymphoblastic leukemia. Blood 114 (13), 2688–2698. 10.1182/blood-2009-03-208397 19641190

[B33] Russell L. J. Jones L. Enshaei A. Tonin S. Ryan S. L. Eswaran J. (2017). Characterisation of the genomic landscape ofCRLF2-rearranged acute lymphoblastic leukemia. Genes, chromosomes cancer 56 (5), 363–372. 10.1002/gcc.22439 28033648 PMC5396319

[B34] Sayyab S. Lundmark A. Larsson M. Ringnér M. Nystedt S. Marincevic-Zuniga Y. (2021). Mutational patterns and clonal evolution from diagnosis to relapse in pediatric acute lymphoblastic leukemia. Sci. Rep. 11 (1), 15988. 10.1038/s41598-021-95109-0 34362951 PMC8346595

[B36] Shochat C. Bandapalli O. R. Palmi C. Ganmore I. te Kronnie G. (2011). Gain-of-function mutations in interleukin-7 receptor-α (IL7R) in childhood acute lymphoblastic leukemias. J. Exp. Med. 208 (5), 901–908. 10.1084/jem.20110580 21536738 PMC3092356

[B37] Stanulla M. Cavé H. Moorman A. V. (2020). IKZF1 deletions in pediatric acute lymphoblastic leukemia: still a poor prognostic marker? Blood 135 (4), 252–260. 10.1182/blood.2019000813 31821407 PMC7019035

[B38] Steeghs E. M. P. Boer J. M. Hoogkamer A. Q. Boeree A. de Haas V. de Groot-Kruseman H. A. (2019). Copy number alterations in B-cell development genes, drug resistance, and clinical outcome in pediatric B-cell precursor acute lymphoblastic leukemia. Sci. Rep. 9 (1), 4634. 10.1038/s41598-019-41078-4 30874617 PMC6420659

[B39] Tasian S. K. Assad A. Hunter D. S. Du Y. Loh M. L. (2018). A phase 2 study of ruxolitinib with chemotherapy in children with Philadelphia chromosome-like acute lymphoblastic leukemia (INCB18424-269/AALL1521): dose-finding results from the part 1 safety phase. Blood 132 (1), 555. 10.1182/blood-2018-99-110221 30093383

[B40] Terwilliger T. Abdul-Hay M. (2017). Acute lymphoblastic leukemia: a comprehensive review and 2017 update. Blood cancer J. 7 (6), e577. 10.1038/bcj.2017.53 28665419 PMC5520400

[B41] Vesely C. Frech C. Eckert C. Cario G. Mecklenbräuker A. Zur Stadt U. (2016). Genomic and transcriptional landscape of P2RY8-CRLF2-positive childhood acute lymphoblastic leukemia. Leukemia 31 (7), 1491–1501. 10.1038/leu.2016.365 27899802 PMC5508072

[B43] Wang Y. Li J. Xue T. L. Tian S. Yue Z. X. Liu S. G. (2023). Clinical, biological, and outcome features of P2RY8-CRLF2 and CRLF2 over-expression in pediatric B-cell precursor acute lymphoblastic leukemia according to the CCLG-ALL 2008 and 2018 protocol. Eur. J. Haematol. 110 (6), 669–679. 10.1111/ejh.13948 36814093

[B44] Yu X. Guo W. H. Lin H. Cheng R. Monroy E. Y. Jin F. (2022). Discovery of a potent BTK and IKZF1/3 triple degrader through reversible covalent BTK PROTAC development. Curr. Res. Chem. Biol. 2 (100029), 100029. 10.1016/j.crchbi.2022.100029 36712232 PMC9879287

