# Supplementary figures and images for: Comprehensive detection of CRLF2 alterations in acute lymphoblastic leukemia: a rapid and accurate novel approach
Source: Front Mol Biosci. 2024 Feb 2;11:1362081. doi: 10.3389/fmolb.2024.1362081 (PMC10869515; doi:10.3389/fmolb.2024.1362081)

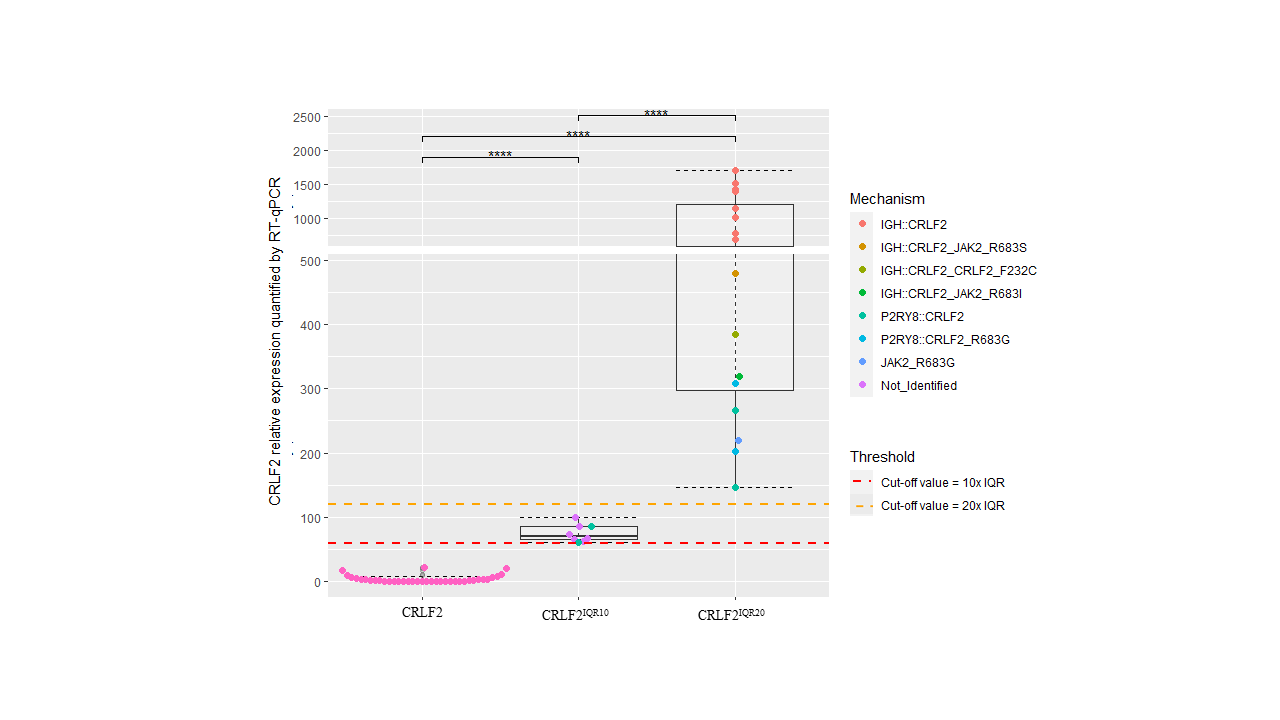

Supplement: Supplementary file 1 [file Image2.PNG]

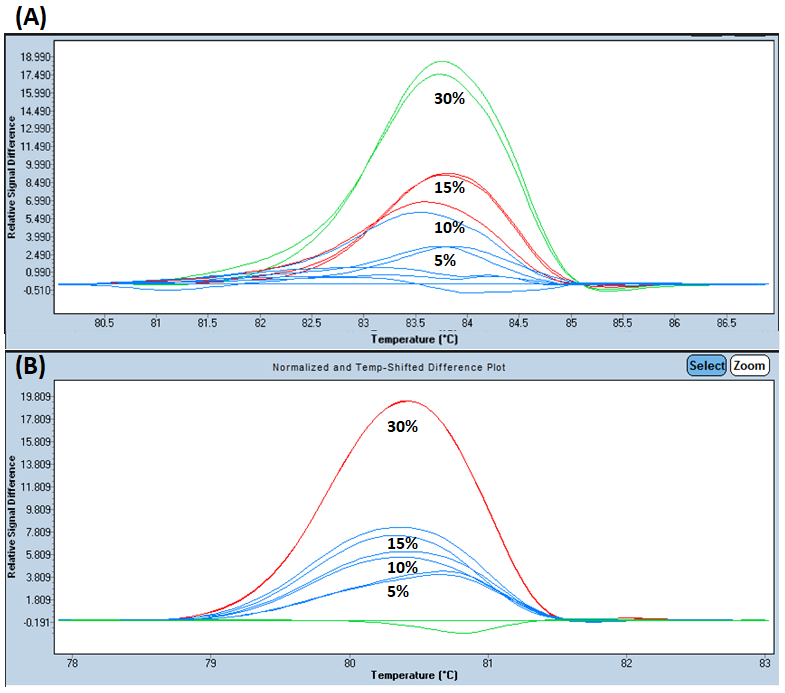

Supplement: Supplementary file 3 [file Image1.PNG]
