# Supplementary material for: Comprehensive detection of CRLF2 alterations in acute lymphoblastic leukemia: a rapid and accurate novel approach
Source: Front Mol Biosci. 2024 Feb 2;11:1362081. doi: 10.3389/fmolb.2024.1362081 (PMC10869515; doi:10.3389/fmolb.2024.1362081)
Supplement: Supplementary file 4 [file DataSheet1.docx]

Supplementary Material
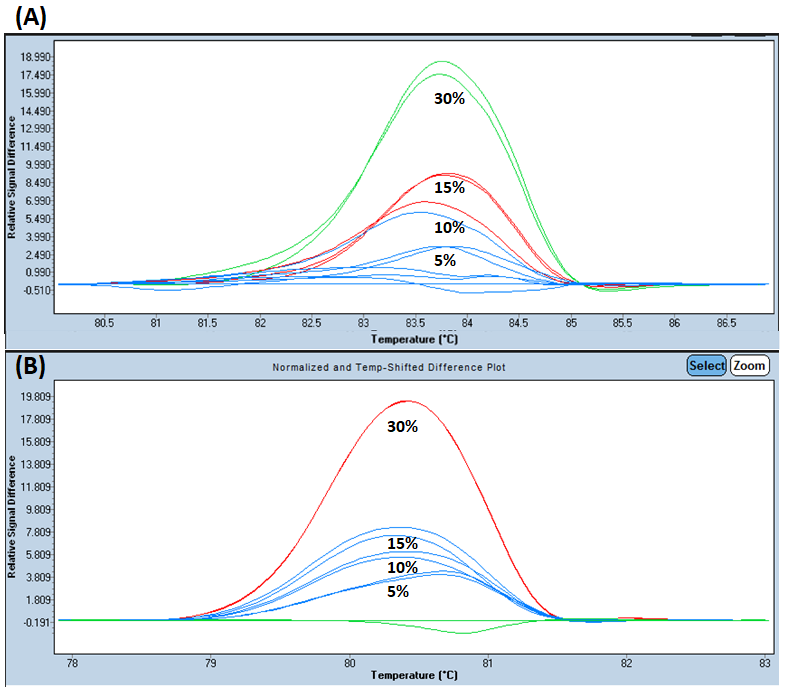


Supplementary Figure 1. Limit of detection of HRM analysis for exons 6 and 16 CRLF2 and JAK2 mutations, respectively. Differential plots obtained with the exon 6 mutation p.F232C for CRLF2 (A) and exon 16 mutation p.R683G for JAK2 (B) serially diluted in wild-type DNA.


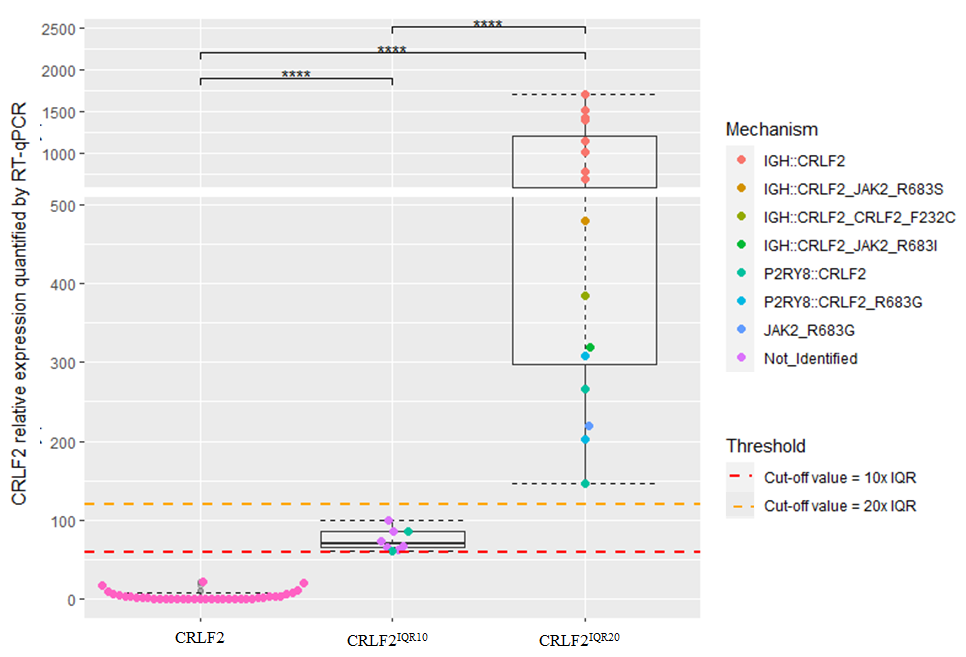


**Supplementary Figure 2.** Box and Whisker plots depicting *CRLF2* expression. Patients were categorized into three distinct groups based on their expression levels: *CRLF2*, *CRLF2*^IQR10^, and *CRLF2*^IQR20^. *CRLF2*^IQR20^ and *CRLF2*^IQR10^ cutoff values are indicated in orange and red dashed lines, respectively. Color-coded dots represent identified underlying molecular mechanisms. **** indicate a p-value <0.001.
